# Prevalence of suicidal ideation and attempted suicide in the general population of Ethiopia: a systematic review and meta-analysis

**DOI:** 10.1186/s13033-021-00449-z

**Published:** 2021-03-24

**Authors:** Berhanu Boru Bifftu, Bewket Tadesse Tiruneh, Berihun Assefa Dachew, Yonas Deressa Guracho

**Affiliations:** 1 School of Nursing, University of Gondar College of Medicine and Health Science, Gondar, Ethiopia; 2 Department of Psychiatry, College of Medicine and Health Science, Bahir Dar University, Bahir Dar, Ethiopia; 3 Department of Epidemiology and Biostatistics, Institute of Public Health, College of Medicine and Health Sciences, University of Gondar, Gondar, Ethiopia; 4 Institute for Social Science Research, The University of Queensland, Brisbane, QLD. 4068 Australia

**Keywords:** Ethiopia, Suicide, Attempted suicide, Suicidal ideation

## Abstract

**Background:**

In Ethiopia, in spite of the high burden of suicide related-adverse effect, substantial variability in the reported prevalence of individual studies about suicidal ideation and attempted suicide; there is no national level epidemiological evidence. Thus, the present study aimed to determine the pooled prevalence of suicide ideation and suicidal attempt in the general population.

**Methods:**

We followed the PRISMA Guidelines to report the results of the finding. Databases including: PubMed/Medline, SCOPUS, CINAHL (EBSCOhost), African Journal Online (AJOL) and African Indexed Medicus (AIM) were searched. Heterogeneity was assessed by Cochrane chi-square (χ^2^) and quantified by I^2^ statistics test. Sensitivity test and subgroup analysis performed. Publication bias was tested by funnel plots and Egger’s test. Effect size was calculated by random effects model.

**Results:**

A total of 12 studies for suicidal ideation and 10 studies for attempted suicide were included in the study. The prevalence of suicidal ideation and attempted suicide were ranged from 1 to 55% and 0.6% to 14% respectively. The 12-month pooled prevalence of suicidal ideation and suicidal attempt were 9% (5–16%), I^2^ = 99.64%, p < 0.001 and 4% (1–8%), I^2^ = 98.11%, p < 0.001] respectively. The lifetime pooled prevalence of attempted suicide was found to be 4% (3–6%). We found evidence of significant heterogeneity for suicidal ideation [I^2^ = 99.64%, p < 0.001] and attempted suicide [I^2^ = 98.11%, p < 0.001]. The subgroup analysis could not identified source of heterogeneity. The sensitivity analysis showed that none of the point estimates was outside of the overall 95%CI for suicidal ideation and attempted suicide. No evidence of publication bias from the visual inspection of the funnel plot for suicidal ideation and [Egger’s test (P = 0.174)] and attempted suicide [Egger’s test (P = 0.318)].

**Conclusion:**

High prevalence of suicidal ideation and attempted suicide were observed in the general population of Ethiopia. These suggest the need of strengthening the awareness of suicidal behaviours and evaluate the effectiveness of the national health strategy in addressing the issues of suicidal behaviours.

## Background

Suicide is serious public health problem [1–3] that has multiple, complex and inter-related psychological, social and economical impacts across the population [4]. Globally, one million people died by suicide per year (one person every 40 s) [2, 5–8]. It contributes to 1.4% of the global burden of disease and the second cause of death for individuals age between 15 and 29 years [9, 10]. The World Health Organization (WHO) Mental Health Action Plan 2013 to 2020 aims to reduce on the full range of suicidal phenomena including: suicidal ideation and suicidal behaviour by 10% [11]. Suicidal ideation and attempts suicide were the primary risk for ended individual’s life [1, 2, 8, 12]. Evidence showed that 50 to 75% of suicidal ideation and attempts suicide were reported to primary health care prior to took his/her life [13–15]. The global lifetime prevalence of suicidal ideation in the general population was ranged from 3.1 to 56% [10]. The corresponding figures in Africa and Ethiopia were 18.6–20.7 and 6% to 55% [4, 12–18, 18–23] respectively. Similarly, the prevalence of attempted suicide was ranged from 0.4 to 5.1% globally [10, 24, 25], 18.6–20.7 in Africa and 1.4% to 19% in Ethiopia [4, 12–22].

By its nature, suicide is a complex phenomenon that involves the interactions of factors at different level such as: system level (access to means of suicide and health care), individual level (demographic features: female gender, younger age, lower education level, unmarried status) and community level (stigma and discrimination) [4, 26]. For example, systematic reviews and meta-analysis showed factors associated with suicidal behaviours include: psychological, psychiatric, biological and stressful life events [4, 27, 28]. Recently published systematic reviews and meta-analysis among the general population in Europe showed that female gender, age over 65 years, unemployment, low social support, adulthood adversity, childhood adversity, family history of mental disorder, affective disorder, major depression, anxiety disorders and substance use were associated with attempted suicide [27, 28]. In addition to these, the lack of standardized methods for data collection, the legal status of suicide and its impact on reporting, lack of resources, stigma, cultural criticism [2, 7, 8, 23, 29–31] and the association of suicide with a criminal act and insurance policies have been affected the true figure of suicidal behaviours in the general population and compared its extent to an iceberg, where only the tip is visible; while the majority hidden under the surface [4, 10].

In Ethiopia despite several inconsistent individual studies with ranged lifetime prevalence of suicidal ideation (1% to 55%), attempted suicide (0.6% to 14%) [17, 32–39], death by suicide (7.2 to 8.4 per 100,000) [40] and lack of accessible health care facilities, skilled man power, stigma, taboo and cultural criticism toward people with suicidal behaviours; priorities are given for other cause of death like infectious related death and the issue of national level epidemiological evidence of death by suicide has little attention. The contextual, epidemiological understanding of suicidal ideation and attempted suicide is an important first step to design culture specific suicide interventions. Therefore, the present systematic review and meta-analysis aimed to determine the pooled prevalence of suicidal ideation and attempted suicide in the general population of Ethiopia.

## Methods

### Study protocol registration and publication

This systematic review and meta-analysis was conducted according to a priori registered (CRD42018112836) and published protocol [41] with slight modification to the original designed published proposal. This modification is (i) related to the populations (i. e clinical, general population, high school and university students) and (ii) suicide-related outcomes (as suicide morbidity and suicide mortality). Because of the distinct characteristics of clinical patients and students from the general populations and suicide mortality as one end of suicidal behaviours from suicide morbidity as the other end, in the present study, we presented a separate manuscript for the general population and suicide morbidity (suicidal ideation and attempted suicide).

The findings in this report presented within, the first domain of suicide prevention activity [universal activities or interventions] that targeted the general public that has not been identified on the basis of individual risk [42, 43]. According to a framework of Gordon’s continuum of care model, prevention, treatment and maintenance were the three model of care [44, 45]. These are (i) universal activities or interventions, the focus of this study that targeted the general public has not been identified on the basis of individual risk, (ii) selected or targeted interventions that aim to reduce the risk among specific high-risk groups and (iii) indicated interventions include the treatments of targeted individuals presenting with suicidal behaviour. This model was previously adopted to guide identification of population groups and individuals with differing prevention needs, and alignment of these needs with appropriate policies and programmes for suicide prevention [45]. Results were reported in accordance with the Preferred Reporting Items for Systematic Review and Meta-Analysis (PRISMA) [46].

### Search strategy

A compressive search of electronic databases: PubMed/Medline, SCOPUS, CINAHL (EBSCOhost), African Journal Online (AJOL) and African Indexed Medicus (AIM) were searched using keywords, Medical Subject Headings (MeSH) terms. Search strings were established using “AND” and/“OR” Boolean operators for each databases. Moreover, to avoid missing of relevant studies like preprints and grey literatures, web of science (Google and Google Scholars) searched. Finally, reference lists of included articles were searched. All searches were performed until November, 20, 2020. Sample search query for PubMed/MEDLINE: ((suicide [MeSH Terms]) OR (suicide) OR (“suicidal behavior” [MeSH Terms]) OR (“suicidal behavior”) OR (“suicide attempt” [MeSH Terms]) OR (“suicide attempt”) OR (“suicide thoughts” [MeSH Terms]) OR (“suicide thoughts”) OR (“suicide ideation” [MeSH Terms]) OR (“suicide ideation”)) AND (("associated factors" [MeSH Terms]) OR ("associated factors") OR "risk factor" [MeSH Terms]) OR ("risk factors") OR (risk [MeSH Terms]) OR (risk) OR ("determinant factors" [MeSH Terms]) OR ("determinants factors")) AND ((epidemiology [MeSH Terms]) OR (epidemiology) OR (prevalence [MeSH Terms]) OR (prevalence) OR (incidence [MeSH Terms]) OR (incidence)) AND ((“general population” [MeSH Terms]) OR (“general population”) OR (community [MeSH Terms]) OR (community)) AND Ethiopia. Similarly, Scopus, and CINAHL (EBSCO host) databases were searched using similar search terms tailored to each database. There is no language and publication year restriction applied.

### Selection of studies

All studies retrieved through search strategy were imported in to EndNote X7 (Thomson Reuters, New York, USA) and removed duplicated studies. The study selection process had three stages: the first stage was screened based on the title and abstract of retrieved references against the outcome. The second stage was based on the full-text to determine its applicability to the study aim/data’s clarity. Finally, studies were screened based on the eligibility criteria. Two reviewers (YDG and BBB) independently screened the studies. Disagreements were solved by discussion.

### Definition of concepts

Suicidal ideation refers to thoughts, fantasies and wishes about ending of one’s own life [1, 29, 47]. Overlapping terms in the concept of suicide attempt: (i) attempted suicide/self-harm/suicidal intent, refer to a deliberated direct destruction of body tissues with a conscious suicidal intent, (ii) non-suicidal attempt/self-injury, refer to a deliberated direct destruction of body tissues without a conscious suicidal intent [4, 11] and (iii) para-suicide, is a term that does not refer to the intention and covers behaviors that can vary from suicidal gestures to serious attempted suicide but unsuccessful to kill oneself [48]. Most standard guidelines and evidence focus on self-harm irrespective of the intent. In line with other similar studies, in this study, suicidal attempt was defined irrespective of its intent and assessment tools [4, 11, 48]. In the current study, for the assessment of suicidal ideation and attempted suicide, (i) majority (75% of ideation and 90% of attempted) of the included studies used Composite International Diagnostic Interview (CIDI). In Ethiopia, CIDI had established reliability and acceptability [26]. In CIDI, a hierarchy of single item questions used for the assessment of suicidal behaviours: For example, suicidal ideation was assessed by the statement: “Have you thought of taking your life in the past 12 months?”; suicide plan (asked of those who had suicidal ideation): “Did you ever make a plan for taking your own life at any time in the past 12 months?”; attempted suicide (asked to those who answered they had ideation or a plan): “Have you attempted to take your own life in the past 12 months?”, (ii) The remaining 25% of suicidal ideation was assessed by Self-Report Questionnaire-20 (n = 3). SRQ-20 had established reliability and acceptability test in Ethiopia as a screening tool to identify probable common mental disorders. The scale contains an item on suicidal thoughts. Similarly, the remaining 10% of attempted suicide used Suicidal Behaviour Questionnaire-Revised (n = 1).

### Eligibility criteria

According to the PICOS acronym, the following inclusion criteria were used: Participants (P): all studies carried out among the general population, Intervention (I): not applicable, Comparison (C): not applicable, Outcomes (O): prevalence of suicidal ideation and suicidal attempt, Study design (S): observational studies (cross-sectional and cohort/longitudinal) were included and Study setting: all studies carried out in Ethiopia were included in the study. Exclusion criteria were: (1) case reports, conference, reviews and abstracts were excluded (2). Studies conducted among clinical population (e.g. psychiatric, physical problems including HIV/AIDs), students and specific population group such as: one sex, prisoners, refugees and street (homeless), (3) For those studies reporting the overall results of suicidal behavior and not reported separately for suicidal ideation and suicidal attempt and (4) studies not conducted in Ethiopia were excluded.

### Data extraction

Data were extracted from the eligible studies using a pre-conceived and piloted data extraction Microsoft Excel format. Data were simultaneously extracted by two independent reviewers (BBB and YDG). The included data items from each article include: name of the first author, year of publication, study area, study design, data collection tool, sample size and number of cases/prevalence including for each sex.

### Quality assessment

The quality of each article was evaluated using the Joanna Briggs Institute (JBI) quality appraisal criteria adapted for reporting the prevalence data for cross-sectional studies [49]. This JBI quality assessment tool has nine items with response option of “yes”, “no”, “unclear” and “not applicable”. These items include: sample frame, sampled participants, sample size, description of study subjects and setting in details, data analysis with sufficient coverage of the identified sample, use of valid method in the identification of conditions, measurements of outcome with a standard, reliable way, appropriateness of statistical analysis and the adequate of response rate. The overall quality of each study was determined by the summed score of all items to generate a study specific global score. Studies were included in analysis if the sum of the overall individual study score is ≥ 50% of the quality assessment checklist criteria’s. Finally, based on the overall mean score (8) we categorized individual study as high quality (≥ 8) and low quality (< 8). Two reviewers (YDG and BBB) independently screened the studies. Disagreements were solved by discussion.

### Data synthesis and statistical analysis

The extracted data into Microsoft Excel Database were exported into Stata 14 that we installed packages for meta-analyses online. Metaprop command in Stata employed to calculate the pool point prevalence estimate with 95% CIs by a random-effects model [50] using the Dersimonian and Laird transformed inverse variance method [51]. Test for Heterogeneity was performed using Cochrane chi-square (χ^2^) and the *I*^2^ statistics. I^2^ value greater than 50% was considered as indicative of substantial heterogeneity [52]. Two separate meta-analyses were performed for the overall outcome (suicidal ideation and suicidal attempt). To assess the possible source of heterogeneity, sensitivity analyses [53] and subgroup analyses by sex, sample size, study setting, publication years and time frame were conducted. Publication bias was tested by the visual inspection of funnel plot [54] and egger test [55]. A p-value < 0.1 was considered as indicative of statistically significant publication bias. The findings of this study summarized and presented using texts, forest plots, tables and summery of descriptive statistics.

## Results

**Fig. 1:**
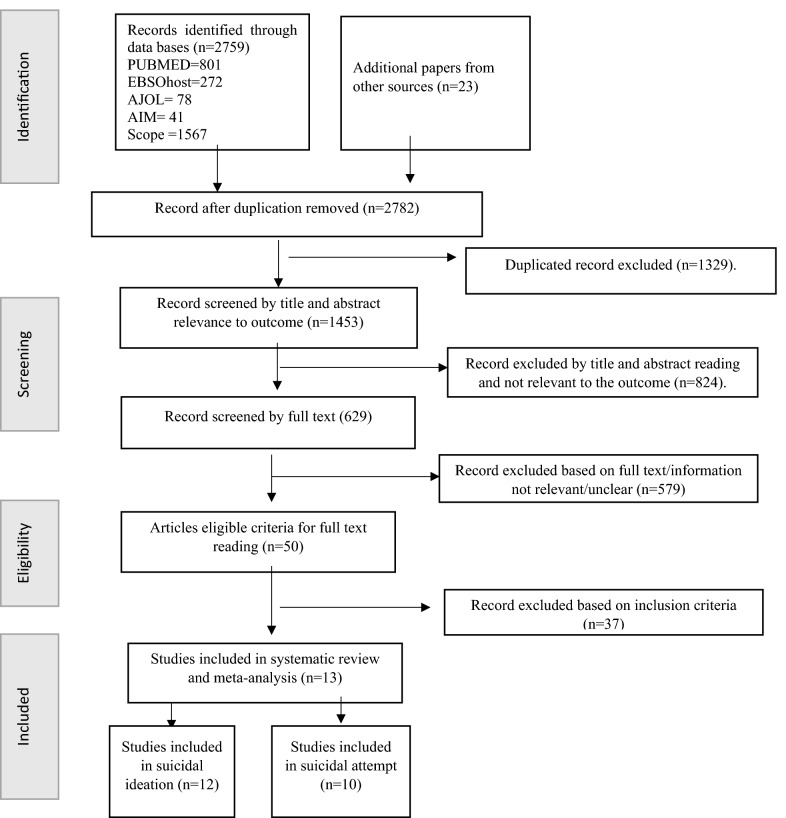
Flow diagram of included studies

The initial database search resulted in 2759 publications. Additional, 23 studies were located from the other sources. This resulted in 2782 records. Of these records, 1329 were excluded because of duplication. Of the remaining 1453, 824 studies were excluded by their title and abstract reading which were not related to the outcome and 579 articles were not full text. Finally, 37 publications were excluded based on the eligibility criteria and objective (Fig 1).

### Study characteristics

In this study, a total of 13 studies were included in the systematic review and meta-analysis. Of these, 12 studies reported suicidal ideation and 10 studies attempted suicide. These studies were carried out in five different regions of the country: South Nation and Nationality People (SNNP) (n = 5), Addis Ababa (n = 2), Oromia (n = 2) and Harar (n = 1) and Tigray (n = 1). All studies used cross-sectional study design. The sample sizes of included studies ranged from 351 to 10,203 for suicidal ideation and 351 to 10,468 for attempted suicide. Studies were published between 1999 and 2020. For the assessment of suicidal ideation, the vast majority (75%) used the Composite International Diagnostic Interview (CIDI) and the remaining three studies used Self-Report Questionnaire 20 (SRQ-20). Similarly, 90% of attempted suicide was assessed by CIDI and the remaining one study used Suicidal Behaviour Questionnaire-Revised (SBQ-R) (Table 1).

**Table 1:** Characteristics of included studies

Author	Year	Setting	population	tools	Time frame	Total	Case (percent)	
							Ideation	Attempt
Jordan	2017	SNNP	Community	CIDI	12-month	1486	104 (7)	55 (3.7)
Fekadu	2016	SNNP	Community	CIDI	12-month	1484	93 (6)	56 (4)
Fekadu	2014	SNNP	Community	CIDI	12-month	1497	202 (13)	21 (1.4)
Fekadu	2007	SNNP	Community	CIDI	12-month	1504	829 (55)	221 (14.7)
Fekadu	2008	Oromia	Community	CIDI	12-month	351	125 (36)	18 (5)
Alem A	1999	SNNP	Community	CIDI	Lifetime	10,468	314 (3)	332 (3.2)
Kebede D	1999	Addis Ababa	Community	CIDI	Lifetime	10,203	277 (3)	275 (2.7)
Nyundo	2019	Oromia	Community	CIDI	12-month	1059	64 (6)	38 (4)
Nyundo	2019	Harar	Community	CIDI	12-month	951	11 (1)	6 (0.6)
Mekonen	2020	Addis Ababa	Community	SBQ-R	Lifetime	481	–	47(10)
Gelay	2012	Addis Ababa	Community	SRQ-20	12-month	2180	47 (2)	–
Hunduma	2017	Harar	Community	SRQ-20	12-month	901	93(10.3)	–
Abbay	2018	Tigray	Community	SRQ-20	12-month	260	12(4.6)	–

*CIDI* Composite International Diagnostic Interview, *SBQ-R* Suicidal Behaviour Questionnaire-Revised and, *SRQ-20* Self-Report Questionnaire 20, *SNNP* South Nation and Nationality People

### Quality of included studies

The quality of included articles in this systematic review and meta-analysis is shown in Table 2. Eight-five percent of the studies used an appropriate sample frame. All studies (100%) used a standard instrument, described the study subjects, setting and employed appropriate statistical analysis. The list scored quality item was observed in the sample size (31%). The overall quality score of included studies had a mean score of 7.6 (ranged 7–9). Ten articles (77%) were high-quality and three studies were low quality.

**Table 2:** JBI Critical Appraisal Checklist for the included studies in the analysis

Authors	Year	Quality domain									Overall Score
		Q1	Q2	Q3	Q4	Q5	Q6	Q7	Q8	Q9	
Jordan	2017	Y	Y	Y	Y	Y	Y	Y	Y	N	8
Fekadu	2016	Y	Y	Y	Y	Y	Y	Y	Y	Y	9
Fekadu	2014	Y	Y	Y	Y	Y	Y	Y	Y	Y	9
Fekadu	2007	Y	Y	Y	Y	Y	Y	Y	Y	Y	8
Fekadu	2008	Y	Y	Y	Y	Y	Y	Y	Y	Y	8
Alem A	1999	Y	Y	Y	Y	Y	N	Y	Y	Y	8
Kebede D	1999	Y	Y	Y	Y	Y	N	Y	Y	Y	8
Nyundo	2019	Y	Y	Y	Y	Y	N	Y	Y	N	7
Nyundo	2019	Y	Y	Y	Y	Y	N	Y	Y	N	7
Mekonen	2020	Y	Y	N	Y	Y	Y	Y	Y	Y	8
Gelay	2012	Y	Y	N	Y	N	Y	Y	Y	Y	7
Hunduma	2017	N	Y	Y	Y	Y	Y	Y	Y	Y	8
Abbay	2018	N	Y	Y	Y	Y	Y	Y	Y	Y	8

A. Q1-Q9 represents questions used to assess the quality of included studies, which are listed below

Q1. Was the sample frame appropriate to address the target populations?

Q2. Were the study participants sampled in appropriate way?

Q3. Was the sample size adequate?

Q4. Were the study subjects and setting described in details?

Q5. Was the data analysis conducted with sufficient coverage of the identified sample?

Q6. Was a valid method used in the identification of conditions?

Q7. Was the condition measured in a standard, reliable way for all participants?

Q8. Was there an appropriate statistical analysis?

Q9. Was the response rate adequate, and if not, was the low response rate managed appropriately?

B. Y, yes; N, no; U, unclear; NA, not applicable

### Prevalence of suicidal ideation and attempted suicide

#### Suicidal ideation

**Fig. 2:**
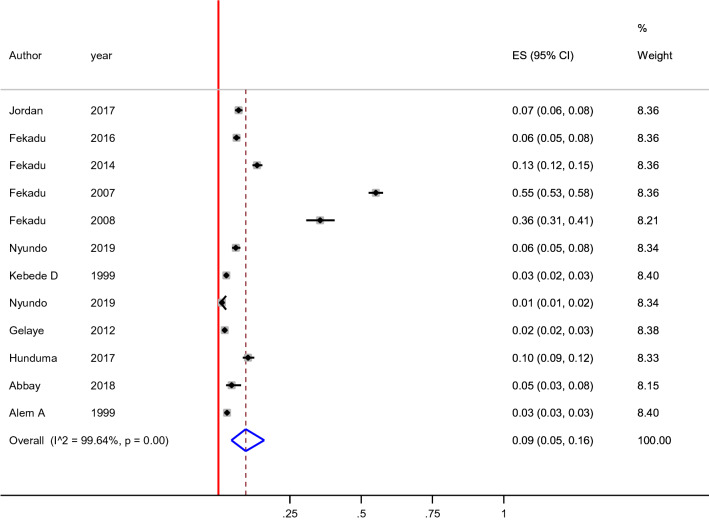
Forest plot presenting 12-month pooled prevalence of suicidal ideation using random effect models with 95% CI

**Fig. 3:**
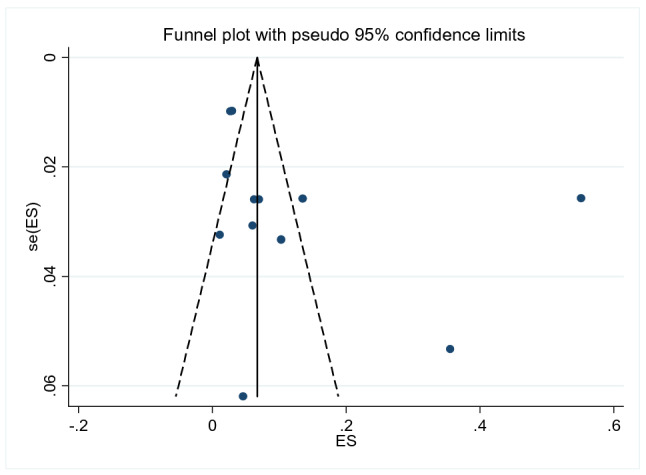
Funnel plot with pseudo 95% CI presented the publication bias for suicidal ideation

The prevalence of suicidal ideation was ranged from 1 to 55% (Table 1). The 12-month pooled prevalence estimate of suicidal ideation was 9% (5%-16%), [I^2^ = 99.64%, p < 0.001, n = 12], (Fig. 2). No lifetime reported prevalence of suicidal ideation.We found evidence of significant heterogeneity [I^2^ = 99.64%, p < 0.001, (Fig. 2)]. The subgroup analysis by study setting, sample size, publication year and quality of study could not identified source of heterogeneity; however, the highest pooled prevalence was considered among studies that carried out between 1999 to 2010 [19% (95% CI 5–39%)] (Table 3). The sensitivity analysis showed that none of the point estimates was outside of the overall 95%CI. No evidence of publication bias from the visual inspection of the funnel plot (Fig. 3) and [Egger’s test (P = 0.174)].

**Table 3:** Subgroup analysis of suicidal ideation and suicidal attempt by study population, sample size, setting, publication year, tool and time frame

Subgroup	Suicidal ideation					Suicidal attempt				
	No. of studies	Pooled prevalence	95% CI	I^2^	p-value	No. of studies	Pooled prevalence	95% CI	I^2^	p-value
Sample size										
Small (< median)	5	9	2–19	98.99%	< 0.001	4	5	1–11	91.16%	< 0.001
Large (≥ median)	7	10	3–19	99.80%	< 0.001	6	3	2–5	98.47%	< 0.001
Study setting										
SNNP	5	14	2–32	99.78%	< 0.001	5	5	2–8	98.74%	< 0.001
Oromia	2	11	10–13	–	–	2	4	3–5	–	–
Addis Ababa	2	3	2–3	–	–	2	3	2–3	–	–
Harar	1	5	4–6	–	–	1	2	1–3	–	–
Tigray	1	5	3–8	–	–	–	–	–	–	–
Publication year										
1999–2010	4	19	5–39	99.89%	< 0.001	5	5	2–8	99.86%	< 0.001
2011–2020	8	6	3–9	97.86%	< 0.001	5	4	2–6	97.58%	< 0.001
Quality of studies										
High	9	14	6–26	99.79%	< 0.001	8	5	3–7	96.14%	< 0.001
Low	3	4	2–8	96.76%	< 0.001	2	2	1–3	–	–
Tool										
CIDI	7	14	4–30	99.68%	< 0.001	7	4	1–8	97.86%	< 0.001
SRQ-20	5	4	3–6	95.54%	< 0.001	–	–	–	–	–
SRB-R	–	–	–	–	–	1	3	2–4	–	–
Timeframe										
12–month	12					7	4	1–8		
Lifetime	–	–	–	–	–	3	4	3–6	97.86%	< 0.001

95% CI represents the 95% Confidence Interval for prevalence and I^2^ true heterogeneity

#### Attempted suicide

**Fig. 4:**
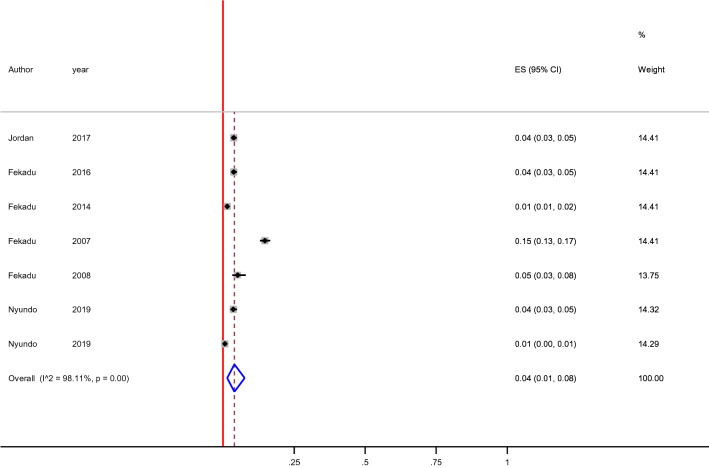
Forest plot presenting 12-month pooled prevalence of suicidal attempt using random effect models with 95% CI

**Fig. 5:**
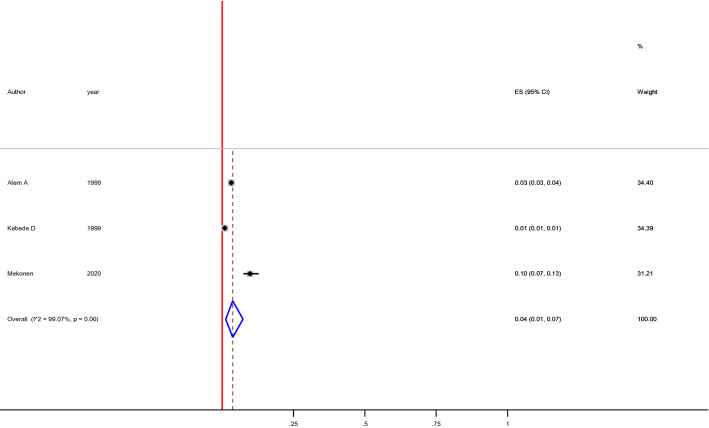
Forest plot presenting lifetime pooled prevalence of suicidal attempt using random effect models with 95% CI

**Fig. 6:**
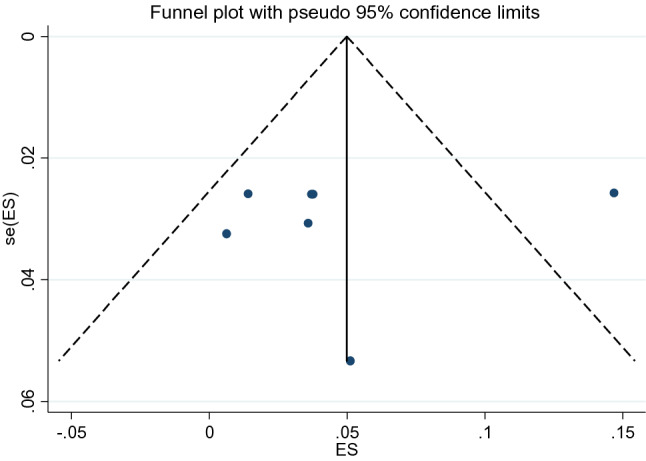
Funnel plot with pseudo 95% CI presented the publication bias for suicidal attempt

In this systematic review and meta-analysis, the prevalence of attempted suicide was ranged from 0. % to 14%. The 12-month and lifetime pooled prevalence of attempted suicide was 4% (1–8%), [I^2^ = 98.11, p < 0.00, n = 10] (Fig. 4) and 4% (1–7%), [I^2^ = 90.07, p < 0.001, n = 3] (Fig. 5) respectively. We found a statistical significant evidence of heterogeneity across studies [I^2^ = 98.11%, p < 0.001, (Fig. 4) for 12-month] and [I^2^ = 99.07, p < 0.001, (Fig. 5) for lifetime]. The subgroup analysis by study setting, sample size, publication year and quality of study could not identified source of heterogeneity; however, the highest pooled prevalence was observed among studies used CIDI assessment tool [14% (95% CI 4–30%)] (Table 3). The sensitivity analysis showed that none of the point estimates was outside of the overall 95%CI. No evidence of publication bias from the visual inspection of the funnel plot (Fig. 6) and [Egger’s test (P = 0.745)].

#### Sex distribution of suicidal ideation and attempted suicide

**Fig. 7:**
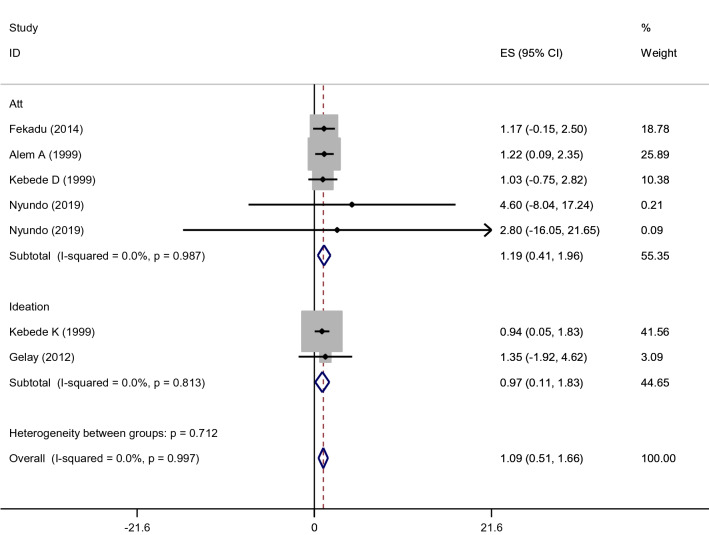
Forest plot presenting the adjusted odds ratios with 95% CIs on the association of females sex with suicidal attempt

Regarding the sex distribution, a total of seven studies (one suicidal ideation and six attempted suicide) reported data on the sex distribution of suicidal behaviours. From this, the sex distribution of suicidal ideation was 2.7% for female and 2.9 for male. On the other hand, the pooled effect of six studies for attempted suicide showed that females were around 1.19 times (AOR = 95%CI 0.41–1.96, I^2^ = 0%, p = 0.987) more likely experienced attempted suicide as compared to males; yet, insignificant (Fig. 7).

## Discussion

To the best of the authors’ knowledge, there is no previous systematic review and meta-analysis has been reported about the pooled prevalence of suicidal ideation and attempted suicide in Ethiopia. The observed prevalence of suicidal ideation [1% to 55%]) and attempted suicide [0.6% to 14%]) in this study is within the reported prevalence of suicidal ideation and attempted suicide across the world [10]. Comparing with other similar studies, the pooled prevalence of suicidal ideation [9% (5–16%), for 12-month] and attempted suicide [(4% (1–8%, for 12-month) and (4% (1–7%), for lifetime] in this study is similar with the 12-month pooled prevalence of suicidal ideation in developing countries (8.1%) and the lifetime pooled prevalence of attempted suicide in Europe [2.88% (2.15%–3.6) and other meta-analysis of community study [6% (4–9%)] [4]. On the other hand, the observed12-month and lifetime pooled prevalence attempted suicide in this study is higher than the pooled prevalence meta-analysis in Europe [(0.57 (0.1–0.6) for 12-month] and [2.88% (2.15–3.6%) for lifetime] [27, 28], China [0.2% (0.1–0.6%) for 12-month] and [0.8% (0.7–0.9%) for lifetime] [56], and lifetime pooled prevalence across 17 countries [2.7%] [26]. The possible explanation for the variation may be due to the difference in educational level, accessibility of information, socio-economic status, and availability of resource, community’s attitude toward suicidal behavior and geographical areas. In Ethiopian, for family with natural death strong social support system has been practiced; yet, fear associated with the stigma, cultural taboo, cultural criticism affect community’s attitude, practice and help seeking behaviours.

The observed high prevalence of attempted suicide in female; yet, insignificant is consistent with study carried out in the general populations of China [57]. This may be attributed to the high prevalence of sexual violence and depression in female as compared to males or they might have the history of abortion which is associated with higher rates of suicide. This is supported by other evidences [58–60]. In Ethiopia, evidence showed the association of sexual violence, early marriage (before 18 years) with suicidal attempt [60]. Another possible explanation may be due to the variation in gender specific cause of suicidal behaviours. For example, a recently published systematic review and meta-analysis showed that eating disorder, posttraumatic stress disorder, bipolar disorder, violence, depressive symptoms and previous history of abortion as female specific risks for suicidal attempts [58, 59]; where, majority of these factors are the major problems in Ethiopia.

Therefore, the finding of this study implies; the existence of high burden of suicidal ideation and suicidal attempt in the general population. This suggests (i) the need of community based screening particularly for those who have difficult to access healthcare facilities (ii) the needs of strengthening community awareness toward suicidal behaviours. Suicide is a tragedy that can be prevented only when one can stand for each other. (iii) at the moment, however, the HMIS does not have a priority indicators for intentional harm, the WHO comprehensive action plan for mental health is suggested to report suicide rate as an urgent priority and (v) suggest the needs of evaluating the effectiveness of the national mental health strategy in addressing suicidal behaviours. This is supported by studies, which showed factors such as absence of psychotic, have unmanageable debts, self-employed, higher levels of social problem solving were associated with the difficult to prevent suicide [13–15, 25].

### Strengths and limitations of the study

This is the first systematic review and meta-analysis about suicidal ideation and attempted suicide in Ethiopia, and use of reference lists and Google Scholar to include all the available studies. However, limitations like exclusion of clinical patient, students, prisoner and the observed heterogeneity in spite of the subgroup analysis may limit the representativeness of the study.

## Conclusion

High prevalence of suicidal ideation and attempted suicide were observed in the general population of Ethiopia. This suggests the need of strengthening the awareness of suicidal behaviours. It also suggests the needs of evaluating the effectiveness of the national health strategy in addressing suicidal behaviours among non-clinical population.

## Data Availability

Not applicable.

## Abbreviations

CI: Confidence interval
CIDI: Composite international diagnostic interview
CRD: Centre for reviews and dissemination
SRQ-20: Self-Report Questionnaire 20
DSH: Deliberate self-harm
DSM-IV: Diagnostic and Statistical Manual of Mental Disorders 4th edition
EJHD: Ethiopian Journal of Health Development
EJHS: Ethiopian Journal of Health Science
EMBASE: ExcerptaMedica database
I^2^: Index of heterogeneity
ICD-10: Tenth Revision of the International Statistical Classification of Diseases and Related Health Problems
MEDLINE: Medical Literature Analysis and Retrieval System Online
MeSH: Medical Subject Headings
NSSI: Non-suicidal self-injury
OR: Odd ratio
PRISMA: Preferred Reporting Items for Systematic Review and Meta-Analysis
PROSPERO: International Prospective Register of Systematic Reviews
SBQ-R: Suicidal Behaviour Questionnaire-Revised
SNNP: South Nation and Nationality People
WHO: World Health Organization
